# Hand Phlegmons: Clinical Burden, Microbiological Spectrum, and Predictors of Surgical Complexity

**DOI:** 10.3390/diagnostics16142184

**Published:** 2026-07-13

**Authors:** Christian Prangenberg, Alberto Alfieri Zellner, Jonas Roos, Lisa Fiona Roder, Johanna Thiele, Amadeo Touet, Kristian Welle, Gunnar Thorben Rembert Hischebeth

**Affiliations:** 1 Department of Orthopedics and Trauma Surgery, University Hospital Bonn, 53127 Bonn, Germany; alberto.zellner@ukbonn.de (A.A.Z.); jonas.roos@ukbonn.de (J.R.); lisa_fiona.roder@ukbonn.de (L.F.R.); johanna.thiele@ukbonn.de (J.T.); amadeo.touet@ukbonn.de (A.T.);; 2 Institute of Medical Microbiology, Immunology and Parasitology, University Hospital Bonn, 53127 Bonn, Germany; gunnar.hischebeth@ukbonn.de

**Keywords:** hand phlegmon, hand infection, surgical debridement, microbiological analysis, Methicillin-resistant *Staphylococcus aureus* (MRSA)

## Abstract

**Background/Objectives:** Hand phlegmons are rapidly progressive bacterial soft-tissue infections that may lead to severe functional impairment, repeated surgical interventions, and prolonged hospitalization if diagnosis and treatment are delayed. Although Gram-positive skin flora are considered the predominant pathogens, microbiological findings and clinical severity may vary considerably depending on the extent of infection and host factors. **Methods:** This single-center retrospective study analyzed 100 patients surgically treated for hand phlegmons at a university hospital between 2020 and 2025. Clinical data included demographics, portal of entry, microbiological findings, inflammatory markers, antibiotic therapy, number of surgical procedures, and length of hospital stay. Pathogens were classified according to Gram stain characteristics, and statistical analyses were performed using nonparametric methods and correlation analyses. **Results:** The cohort consisted of 69 male and 31 female patients with a mean age of 50.5 ± 20.9 years. Mean hospital stay was 12.9 days (range 1–77), and patients underwent an average of 2.18 surgical procedures (range 0–11). Wounds and bite injuries represented the most common portals of entry, although many cases lacked a clearly identifiable cause. Microbiological cultures were negative in 62% of cases. Among positive cultures, Gram-positive organisms predominated (84.2%), with *Staphylococcus aureus* and *Streptococcus pyogenes* representing the most frequent pathogens. MRSA was detected in three patients and was associated with significantly more surgical procedures (*p* = 0.04) and a tendency toward prolonged hospitalization. Admission CRP levels differed significantly according to Gram stain classification (*p* = 0.008) and correlated positively with both length of hospital stay (r = 0.431, *p* < 0.001) and number of surgical interventions (r = 0.272, *p* = 0.006). Patients with microbiologically confirmed infections required significantly more operative procedures than patients without pathogen detection (*p* < 0.001). Empiric antibiotic therapy primarily consisted of ampicillin/sulbactam, cefuroxime, and clindamycin, followed by oral therapy with amoxicillin–clavulanate or clindamycin. **Conclusions:** Hand phlegmons remain clinically challenging infections characterized by heterogeneous presentation, frequent culture-negative findings, and substantial surgical burden. Gram-positive pathogens continue to predominate, while resistant organisms such as MRSA may be associated with more complex clinical courses. Elevated inflammatory markers at admission correlate with prolonged hospitalization and increased operative requirements, suggesting their potential value as indicators of disease severity. Early diagnosis, prompt surgical debridement, and individualized antimicrobial therapy remain essential for infection control and preservation of hand function.

## 1. Introduction

Hand phlegmons represent rapidly progressive bacterial soft-tissue infections that constitute a serious threat to hand function and, in advanced cases, to overall patient health if diagnosis and treatment are delayed [1]. Even minor skin injuries such as puncture wounds, small lacerations, bites, or abrasions can serve as portals of entry, allowing infection to spread along tendon sheaths and fascial planes into deep anatomical compartments of the hand [2,3,4]. Owing to the complex anatomy of the hand and its limited tolerance for swelling, early recognition and prompt intervention are essential to prevent irreversible functional impairment and systemic complications [5,6].

From a microbiological perspective, hand phlegmons are caused by a heterogeneous spectrum of pathogens. Gram-positive organisms, particularly *Staphylococcus aureus* and *Streptococcus* species, predominate in acute infection [1,7,8]. Increasing rates of methicillin-resistant *Staphylococcus aureus* (MRSA) have been documented, especially in urban and healthcare-associated settings, influencing empiric antibiotic strategies [9]. In selected cases—such as delayed presentation, chronic wounds, bite injuries, or immunocompromised patients—Gram-negative bacteria, anaerobes, or polymicrobial infections may be encountered, underscoring the importance of microbiological assessment and culture-guided therapy [8,10]. Certain patient populations, including individuals with diabetes mellitus, peripheral vascular disease, renal insufficiency, or immunosuppression, are at increased risk for severe infections, prolonged hospitalization, and poorer functional outcomes [11].

**Figure 1:**
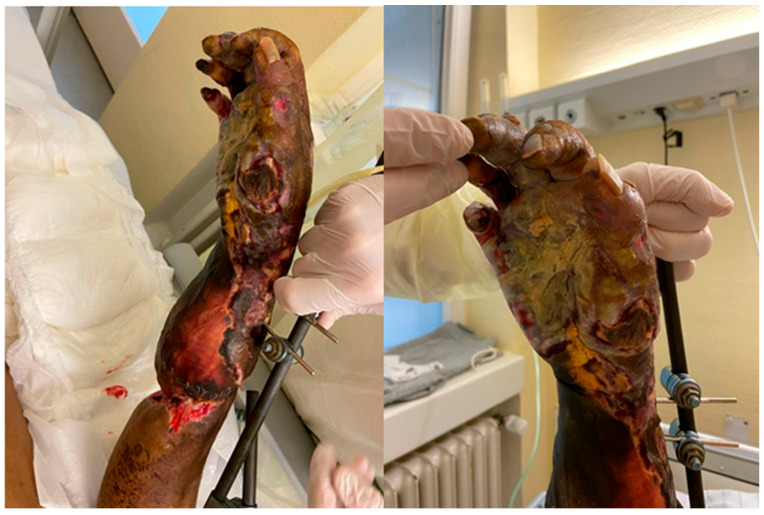
Advanced hand phlegmon following an open distal radius fracture with confirmed methicillin-resistant Staphylococcus aureus (MRSA) infection. The two images illustrate the extensive soft-tissue involvement resulting from delayed surgical management, as definitive operative treatment was not performed in a timely manner due to limited medical resources during treatment abroad.

The management of hand phlegmons relies on a combined surgical and pharmacological approach. Timely surgical incision, drainage, and debridement remain the cornerstone of treatment and are strongly associated with favorable outcomes and infection resolution [1,5]. Antibiotic therapy serves as an essential adjunct, with empiric regimens guided by local epidemiology and subsequently refined according to culture and susceptibility results [2,4]. Advances in diagnostic imaging, including point-of-care ultrasound, may further support early detection and treatment planning, particularly in emergency settings [12] Figure 1.

This paper aims to present a retrospective analysis of surgically treated hand phlegmons, focusing on clinical presentation, microbiological findings, treatment strategies, and patient outcomes. By evaluating our institutional experience, we seek to identify factors associated with disease severity and surgical management and to contribute to the current understanding of these potentially devastating infections.

## 2. Materials and Methods

This single-center retrospective study included all patients diagnosed and surgically treated for hand phlegmons over a five-year period (1 January 2020, to 31 December 2025) at a university hospital with a specialized hand surgery and trauma unit. The study protocol was approved by the institutional ethics committee (No. 406/17). According to the approval of the local Ethics Committee (No. 406/17), retrospective analysis of routinely collected clinical data was permitted without patient contact or additional informed consent, as no data beyond those obtained during routine clinical care were collected.

Patients were included if they had a clinically and contrast-enhanced computed tomography (CT)-confirmed soft tissue infection of the hand requiring surgical intervention and inpatient treatment. Only patients with soft tissue infections involving the cutis, subcutis, and tendon sheaths were included in this study. Patients with septic arthritis and paronychia were excluded. Surgical treatment generally consisted of incision, drainage, and debridement, with intraoperative tissue samples obtained for microbiological analysis whenever feasible. Patients younger than 18 years and patients with incomplete medical records or missing microbiological data were excluded from the study. Postoperatively, all patients were immobilized with a plaster splint until the infection had resolved. No patient was treated with an external fixator.

Clinical data were extracted from electronic medical records and included demographic characteristics (age and sex), mechanism of injury or documented portal of entry, duration of hospital stay, number of surgical procedures, duration of antibiotic treatment, microbiological findings, and inflammatory laboratory parameters at admission, including leukocyte count and C-reactive protein (CRP).

Two tissue samples were routinely obtained intraoperatively from each patient for microbiological analysis. Microbiological analysis was performed using standard aerobic and anaerobic culture techniques from intraoperatively obtained tissue samples. Pathogens were classified according to microbiological culture results and further categorized based on Gram stain characteristics into no pathogen detected, Gram-positive organisms, Gram-negative organisms, or mixed gram-positive/gram-negative infection. Antibiotic therapy was routinely initiated after intraoperative collection of microbiological specimens with empirical intravenous cefuroxime. Following microbiological results and susceptibility testing, antibiotic therapy was adjusted accordingly and continued intravenously during hospitalization, followed by oral treatment after discharge until complete wound healing. Patients were considered eligible for hospital discharge once the wound was dry, inflammatory markers demonstrated a declining trend, and the infection was clinically controlled. At the time of discharge, intravenous antibiotic therapy was transitioned to oral antibiotics, with the regimen selected according to microbiological findings and the patient’s clinical course. Statistical analysis was performed using descriptive and inferential methods IBM SPSS Statistics 25 (Armonk, NY, USA). Continuous variables are presented as mean ± standard deviation or median with range, whereas categorical variables are reported as frequencies and percentages. Because several variables showed non-normal distributions, nonparametric statistical methods were applied. Group comparisons were performed using the Kruskal–Wallis test for multiple independent groups and the Mann–Whitney U test where appropriate. Correlations between inflammatory markers and clinical outcomes were assessed using Spearman rank correlation coefficients. Statistical significance was defined as *p* < 0.05.

## 3. Results

**Figure 2:**
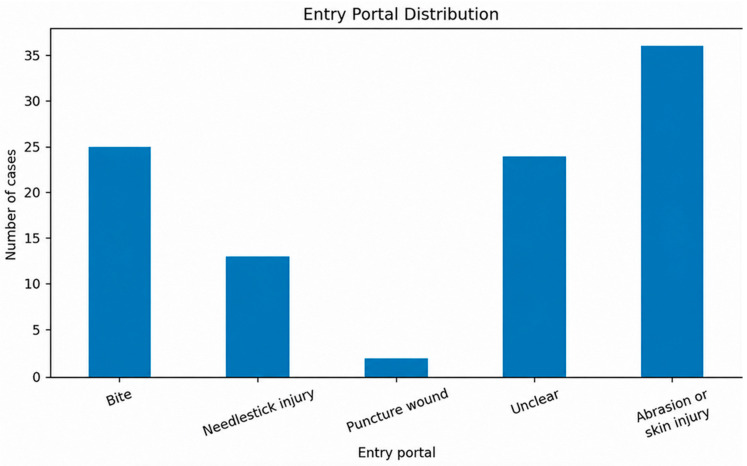
Shows the distribution of entry portals in patients with hand phlegmon. Wounds were the most common entry portal (n = 36), followed by bites (n = 25) and unclear causes (n = 24). Needlestick injuries accounted for 13 cases, whereas puncture wounds were rare (n = 2). Overall, traumatic skin lesions represented the predominant source of infection.

A total of 100 patients were included (69 male, 31 female), with a mean age of 50.5 ± 20.9 years. The mean length of hospital stay was 12.9 days (range 1–77 days), and patients underwent an average of 2.18 surgical procedures (range 0–11). Mechanisms of injury included various documented entry routes, with several cases lacking a clearly identifiable portal of entry (Figure 2).

Microbiological cultures were negative in 62 cases. Positive cultures were identified in 38 patients, with the pathogens detected detailed below Table 1.

**Figure 3:**
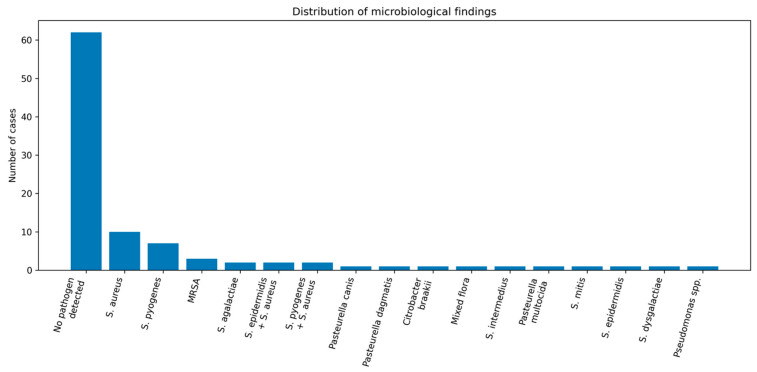
Bacterial findings in hand infections. Most commonly, no pathogen is detected; when present, typical skin bacteria are most frequently identified.

Among positive cultures, Gram-positive organisms predominated (84.2%), whereas Gram-negative organisms accounted for 13.2%, and mixed Gram-positive/Gram-negative flora for 2.6%. This pathogen distribution was consistent with the predominance of common skin flora as the primary source of hand infections (Figure 3).

**Figure 4:**
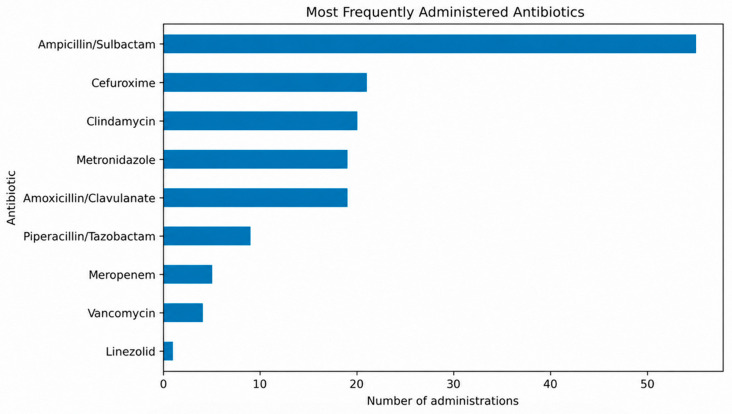
Illustrates the frequency of administered antibiotics in patients with hand phlegmon. Unacid was the most commonly used antibiotic (n = 55), followed by Cefuroxime (n = 21), Clindamycin (n = 20), Metronidazole (n = 19), and Amoxicillin/Clavulanate (n = 19). Broad-spectrum reserve antibiotics such as Piperacillin/Tazobactam, Meropenem, and Vancomycin were used less frequently. The figure highlights the predominance of beta-lactam-based antibiotic therapy in the treatment of hand infections.

The mean duration of antibiotic treatment was 16.5 days (range 1–59 days). Intravenous therapy commonly consisted calculated regimens including ampicillin/sulbactam, cefuroxime, and clindamycin, whereas oral treatment most frequently involved amoxicillin–clavulanate or clindamycin. Combination therapy was administered in selected cases involving polymicrobial infections or resistant organisms (Figure 4).

Surgical management primarily consisted of incision, drainage, and debridement. Repeated surgical interventions were required in a subset of patients, particularly in extensive or deep infections.

Admission CRP levels differed significantly according to Gram stain classification (Kruskal–Wallis H = 11.82, *p* = 0.008). Patients with microbiologically confirmed infections demonstrated significantly higher inflammatory markers compared with patients without pathogen detection, with Gram-positive infections showing the highest CRP levels among the major groups. Mean CRP levels were 39.97 mg/L in patients without pathogen detection, 160.66 mg/L in those with Gram-positive pathogens, and 81.34 mg/L in those with Gram-negative pathogens. The mean leukocyte count at admission was 11.20 × 10^9^/L in patients without pathogen detection, 15.08 × 10^9^/L in patients with Gram-positive infections, and 11.74 × 10^9^/L in patients with Gram-negative infections.

Length of hospital stay also differed significantly according to Gram stain classification (Kruskal–Wallis H = 16.14, *p* = 0.001). Patients with Gram-positive infections had the longest hospitalization periods (mean of 21.9 days). Patients with MRSA had a significantly longer hospital stay compared with all other patients (35.7 vs. 12.2 days; *p* = 0.043). Likewise, patients with *Streptococcus pyogenes* demonstrated a significantly prolonged length of stay compared with patients infected by other pathogens (24.1 vs. 12.1 days; *p* = 0.022). In contrast, the length of stay of patients with *Staphylococcus aureus* did not differ significantly from that of the remaining patient cohort (11.5 vs. 13.4 days; *p* = 0.995). Direct comparisons between *Staphylococcus aureus*, MRSA, and *Streptococcus pyogenes* revealed no significant differences in hospital length of stay. Admission CRP levels showed a moderate positive correlation with length of stay (Spearman r = 0.431, *p* < 0.001), indicating that higher inflammatory activity at presentation was associated with prolonged hospitalization. In contrast, no significant correlation was observed between admission CRP levels and duration of antibiotic treatment (r = 0.146, *p* = 0.150), and antibiotic duration did not differ significantly between Gram stain groups (H = 3.59, *p* = 0.310).

**Figure 5:**
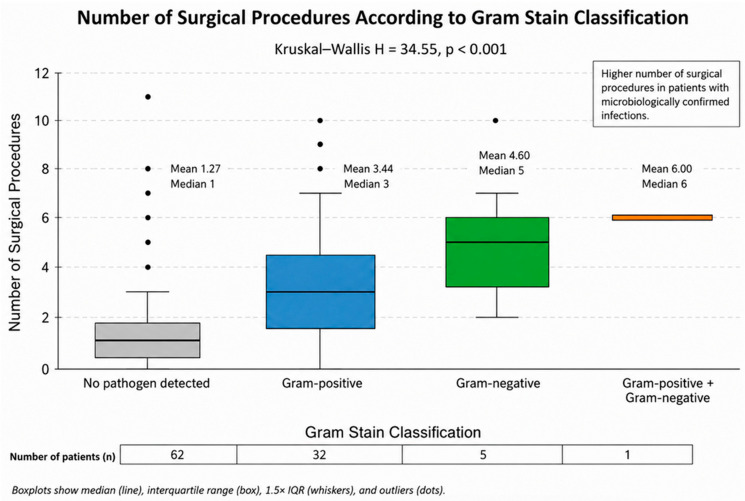
Number of surgical procedures according to Gram stain classification. The boxplots display the median (horizontal line), interquartile range (box), 1.5× interquartile range (whiskers), and outliers (dots). Patients with microbiologically confirmed infections (Gram-positive, Gram-negative, or mixed infections) required significantly more surgical interventions than patients without pathogen detection (Kruskal–Wallis H = 34.55, p < 0.001). Interpretation of the gram-negative and mixed groups is limited due to the small sample size.

The number of surgical procedures differed significantly according to positive microbiological cultures (Kruskal–Wallis H = 34.55, *p* < 0.001), with patients showing microbiologically confirmed infections requiring more operative interventions than those without pathogen detection. All patients underwent surgical debridement followed by routine antiseptic irrigation using Betaisodona solution, hydrogen peroxide (H_2_O_2_), and Ringer’s solution. In patients with severe infections requiring a planned second-look procedure, negative pressure wound therapy was applied. In cases of clinical sepsis, pads were placed instead of negative pressure wound therapy to allow for repeated surgical exploration and adequate drainage. Forty-four patients required only one surgical procedure and underwent primary wound closure after surgical debridement (Figure 5). 

Forty-two patients required more than one surgical procedure. Of these, 28 patients were treated with negative pressure wound therapy (VAC), whereas 14 patients presenting with clinical signs of sepsis underwent temporary packing with 7 pads instead of VAC therapy. Patients with MRSA required significantly more surgical procedures than all other patients (5.3 vs. 2.1 procedures; *p* = 0.044). Similarly, the presence of *Streptococcus pyogenes* was associated with a significantly higher number of surgical interventions (3.4 vs. 2.1 procedures; *p* = 0.006). In contrast, patients with *Staphylococcus aureus* did not undergo significantly more procedures than the remaining patients (2.0 vs. 2.2 procedures; *p* = 0.571). In the direct comparison of the three pathogen groups, patients with *Streptococcus pyogenes* required significantly more surgical procedures than those with *Staphylococcus aureus* (*p* = 0.031), whereas no significant differences were observed between MRSA and either of the other two groups. Furthermore, admission CRP levels demonstrated a weak but significant positive correlation with the number of surgical procedures (Spearman r = 0.272, *p* = 0.006). No significant association was observed between the documented portal of entry and the number of surgical procedures (H = 5.94, *p* = 0.204).

**Figure 6:**
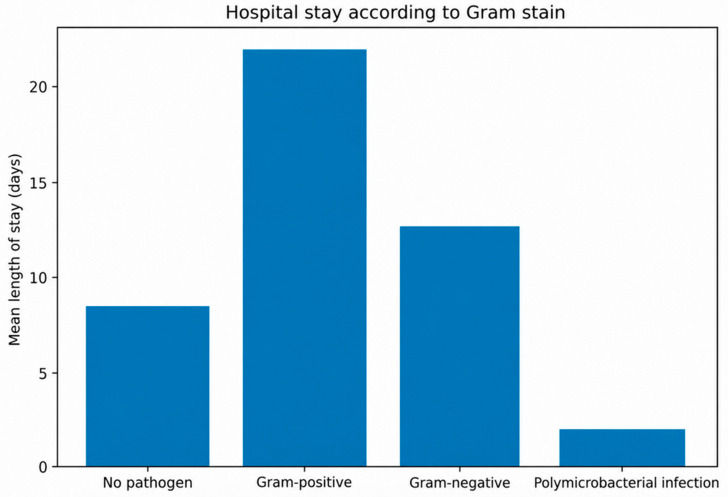
Mean length of hospital stay according to Gram stain classification. Patients with Gram-positive infections showed the longest hospitalization times compared with patients without microbiological pathogen detection. A significant association between Gram stain classification and length of hospital stay was observed (Kruskal–Wallis H = 16.14, p = 0.001). Interpretation of Gram-negative and mixed infections is limited due to the small number of cases.

Patients with MRSA infection showed a tendency toward prolonged hospitalization compared with non-MRSA patients (median 21 vs. 7 days, *p* = 0.08) (Figure 6). 

In addition, MRSA cases underwent significantly more surgical procedures (median 3 vs. 1, *p* = 0.04). Although the number of MRSA cases was limited (n = 3), these findings suggest a more complex clinical course requiring repeated operative interventions.

## 4. Discussion

The present study highlights the clinical complexity of hand phlegmons and underscores the importance of early surgical and antimicrobial management. The patient cohort demonstrated a broad age distribution with a male predominance, which is consistent with epidemiological patterns reported in previous studies on hand infections [6,7,13]. The relatively long mean hospital stay of 12.9 days and the requirement for more than two surgical procedures per patient on average reflect the potentially aggressive nature of these infections and the substantial healthcare burden they impose [6,10,14]. Notably, hospitalization periods varied considerably, ranging from one day to more than two months, illustrating the highly heterogeneous clinical course of hand phlegmons and the potential for prolonged treatment in severe cases.

The mechanisms of injury observed in this cohort were heterogeneous, ranging from bite wounds and abrasions to penetrating and crush injuries, while a considerable proportion of cases lacked a clearly identifiable portal of entry. This finding is in line with previous reports indicating that even minor or unnoticed trauma may precipitate severe hand infections, particularly when early symptoms are underestimated or treatment is delayed [1,4,15]. Importantly, no significant association was observed between the documented portal of entry and the number of surgical procedures required. This suggests that the subsequent severity of infection may depend less on the initial injury mechanism itself and more on factors such as bacterial virulence, host immune response, delayed presentation, or extent of anatomical spread. Bite-associated infections represented a clinically relevant subgroup and were linked to pathogens such as *Pasteurella multocida*, *Pasteurella canis*, and *Pasteurella dagmatis*, organisms classically associated with animal and human bite injuries and known to require specific antimicrobial coverage [2,3].

Accurate microbiological identification is essential for targeted antimicrobial therapy in hand infections. In our cohort, at least two deep tissue specimens were routinely obtained intraoperatively for microbiological analysis. Current international guidelines recommend obtaining deep tissue samples rather than superficial wound swabs whenever feasible, as tissue specimens more accurately reflect the causative pathogens and reduce the risk of contamination by colonizing skin flora. Superficial swabs are associated with lower diagnostic accuracy and may fail to identify the organisms responsible for deep soft tissue infections. Therefore, the routine collection of multiple deep tissue specimens during surgical debridement represents the preferred diagnostic approach and may improve the reliability of microbiological findings and subsequent antibiotic stewardship [16].

Microbiological cultures remained negative in the majority of patients (62%), a phenomenon frequently described in the literature and commonly attributed to prior antibiotic exposure, inadequate tissue sampling, low bacterial burden, or localization of pathogens in deep tissue compartments that are difficult to access microbiologically [1,8,17]. Nevertheless, among positive cultures, Gram-positive organisms clearly predominated (84.2%), supporting the established concept that common skin flora constitute the principal etiological agents in acute hand infections [1,4,7]. *Staphylococcus aureus* and *Streptococcus pyogenes* represented the most frequently isolated pathogens, consistent with previously published bacteriological profiles [1,7,18]. The occurrence of polymicrobial infections and the identification of Gram-negative organisms such as *Citrobacter braakii*, *Klebsiella* spp., *Serratia marcescens*, and *Pseudomonas* spp. underline that severe hand phlegmons may occasionally involve complex mixed flora, especially in bite injuries, delayed presentations, or immunocompromised patients [5,8,17].

The detection of MRSA in three patients deserves particular attention. Although the absolute number was small, MRSA infections were associated with significantly more operative interventions and showed a tendency toward prolonged hospitalization. These findings suggest that resistant organisms may contribute to a more complicated clinical course requiring repeated surgical revision and prolonged inpatient treatment. Similar associations between MRSA infections and increased morbidity have been reported previously in studies of musculoskeletal and soft tissue infections [8,9,19]. Consequently, clinicians should maintain awareness of resistant pathogens, especially in patients with recurrent infections, previous healthcare exposure, or treatment failure under standard empiric regimens.

An important finding of this study was the relationship between inflammatory markers, microbiological findings, and clinical outcome. Admission CRP levels differed significantly according to Gram stain classification, with the highest inflammatory activity observed in Gram-positive infections. Moreover, elevated CRP values correlated moderately with prolonged hospitalization and weakly with the number of required surgical procedures. These findings support the clinical relevance of CRP as a potential surrogate marker for disease severity in hand phlegmons. Although CRP alone cannot reliably predict the microbiological pathogen spectrum, markedly elevated inflammatory markers at presentation may indicate a more extensive infectious process and the likelihood of a prolonged or surgically demanding course. Similar observations have been reported in previous investigations of deep soft tissue infections and septic hand conditions [10,12,14]. Interestingly, no significant association was found between admission CRP levels and duration of antibiotic therapy, nor did antibiotic duration differ significantly between Gram stain groups. This may indicate that the decision regarding antibiotic duration was influenced more strongly by clinical evolution and surgical findings than by microbiological classification alone. In clinical practice, treatment duration is frequently individualized according to wound healing, tissue viability, and postoperative recovery rather than solely based on laboratory parameters [5,13].

The duration and spectrum of antibiotic therapy in this cohort were relatively extensive, with a mean treatment duration of 16.5 days and some patients receiving therapy for up to 59 days. Such prolonged treatment courses exceed durations commonly reported for uncomplicated soft tissue infections but appear justified in extensive deep-space infections, delayed presentation, resistant pathogens, or cases requiring repeated surgical debridement [5,17]. Initial empiric intravenous treatment frequently consisted of broad-spectrum agents such as ampicillin/sulbactam, cefuroxime, and clindamycin, which aligns with current guideline-based recommendations for severe hand infections [2,4,13]. These regimens provide adequate coverage against the predominant Gram-positive pathogens while additionally addressing selected anaerobic and Gram-negative organisms in polymicrobial or bite-associated infections. Subsequent step-down to oral agents such as amoxicillin–clavulanate or clindamycin reflects established antimicrobial stewardship principles once clinical improvement is achieved [8,19]. Combination therapy was selectively used in polymicrobial infections or in cases involving resistant organisms, emphasizing the need for individualized antimicrobial management in complex infections. The duration of antibiotic therapy in our cohort was consistent with current IWGDF/IDSA recommendations for soft tissue infections. Following adequate surgical debridement, the infectious burden was considered substantially reduced, effectively downgrading the infection to a moderate soft tissue infection. Consequently, patients received a total antibiotic course of approximately two weeks, consisting of an initial intravenous regimen followed by oral therapy after clinical improvement. This treatment strategy reflects current guideline recommendations, which support a limited duration of antimicrobial therapy after adequate source control while minimizing unnecessary antibiotic exposure [16].

Surgical intervention remained the cornerstone of treatment in this series (Figure 3). Incision, drainage, and debridement were essential for infection control, and repeated procedures were frequently required in extensive or deep infections. Patients with microbiologically confirmed infections underwent significantly more operative interventions than culture-negative patients, suggesting that pathogen-positive infections may represent a biologically more aggressive subgroup. The significant correlation between CRP levels and number of surgical procedures further supports the association between systemic inflammatory activity and local disease severity. These observations are consistent with earlier studies demonstrating that timely and sufficiently radical surgical management is the most critical determinant of infection resolution and preservation of hand function [1,5,10,15]. Delayed or inadequate debridement may allow continued spread through tendon sheaths, fascial compartments, and deep spaces of the hand, ultimately increasing the risk of stiffness, tissue necrosis, and functional impairment [20,21].

Imaging modalities, particularly ultrasound, may play an increasingly important role in early diagnosis and operative planning, especially in emergency settings where clinical findings are equivocal or deep-space involvement is suspected [3,12]. Advanced imaging techniques may facilitate earlier identification of abscess formation, tendon sheath involvement, or retained foreign bodies and thereby support more targeted surgical intervention. While ultrasound is often sufficient to confirm the presence of a soft tissue infection or fluid collection, contrast-enhanced computed tomography (CT) is frequently required for preoperative planning. In particular, CT allows a more reliable assessment of the extent of the infection, involvement of deep anatomical compartments, and potential osseous involvement. From our perspective, CT therefore represents an essential imaging modality for surgical planning and contributes to the selection of the appropriate operative approach.

**Figure 7:**
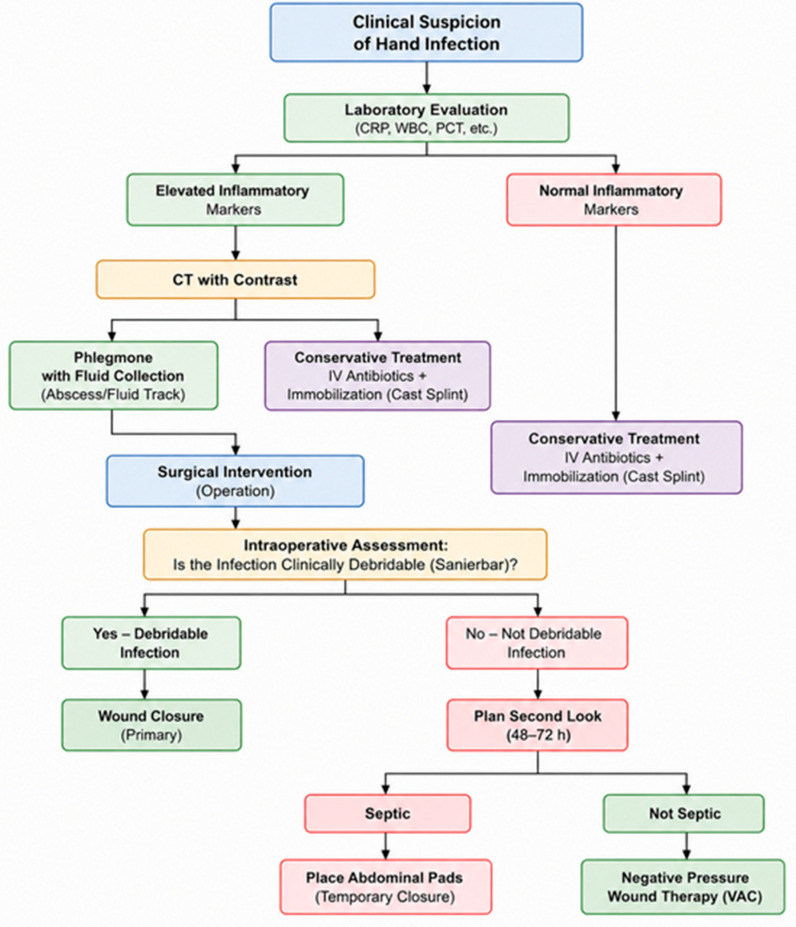
Diagnostic and therapeutic algorithm for the management of suspected hand phlegmon. Patients with elevated inflammatory markers undergo contrast-enhanced CT imaging. Phlegmons with fluid collection are treated surgically, whereas cases without fluid collection are managed conservatively with intravenous antibiotics and immobilization. Intraoperative findings determine whether primary wound closure is feasible or a planned second-look procedure is required. Following second-look surgery, persistent septic wounds are managed with abdominal pad packing, while non-septic wounds receive negative pressure wound therapy (VAC).

Overall, the results of this study reinforce the multifactorial nature of hand phlegmons and the necessity of an integrated diagnostic and therapeutic approach. Based on our findings, we propose a practical diagnostic and therapeutic algorithm for the management of suspected hand phlegmons (Figure 7). 

Clinical suspicion combined with laboratory evidence of infection should prompt contrast-enhanced CT imaging to assess the extent of infection, identify fluid collections, evaluate possible osseous involvement, and facilitate preoperative planning. Patients with fluid collections should undergo timely surgical debridement, whereas those without drainable collections may be managed conservatively with intravenous antibiotics and immobilization. Intraoperative assessment should guide the need for primary wound closure or planned second-look surgery, with subsequent wound management tailored to the septic status. This standardized approach may support clinical decision-making, optimize surgical planning, and contribute to improved patient outcomes. Future prospective studies with larger patient cohorts may further clarify prognostic factors, optimize antimicrobial strategies, and improve risk stratification for patients with severe hand infections [13,14,17].

## Figures and Tables

**Table 1:** Overview of the bacterial species identified in patients with hand phlegmons.

Pathogen	Number of Cases
No pathogen detected	62
*Staphylococcus aureus*	10
*Streptococcus agalactiae*	2
*Streptococcus pyogenes*	7
*Staphylococcus epidermidis* + *Staphylococcus aureus*	2
*Pasteurella canis*	1
*Pasteurella dagmatis*	1
*Citrobacter braakii*	1
Methicillin-resistant *Staphylococcus aureus* (MRSA)	3
Mixed polymicrobial Infection (*Staphylococcus aureus*, *Bacteroides fragilis*, *Serratia marcescens*, *Citrobacter braakii*, *Klebsiella* spp.)	1
*Streptococcus pyogenes* + *Staphylococcus aureus*	2
*Streptococcus intermedius*	1
*Pasteurella multocida*	1
*Streptococcus mitis*	1
*Staphylococcus epidermidis*	1
*Streptococcus dysgalactiae*	1
*Pseudomonas* spp.	1

## Data Availability

The data supporting the findings of this study are not publicly available because they contain sensitive patient information and are protected by applicable data protection regulations. The data are held by the University Hospital Bonn and cannot be made publicly available.
